# Analysis of Population Genetic Diversity and Genetic Structure of *Schizothorax biddulphi* Based on 20 Newly Developed SSR Markers

**DOI:** 10.3389/fgene.2022.908367

**Published:** 2022-06-13

**Authors:** Zhulan Nie, Yongli Ren, Lirong Zhang, Rui Ge, Jie Wei

**Affiliations:** ^1^ College of Animal Science and Technology, Tarim University, Alar, China; ^2^ Key Laboratory of Tarim Animal Husbandry and Science Technology of Xinjiang Production and Construction Corps., Alar, China; ^3^ State Key Laboratory Breeding Base of Protection and Utilization of Biological Resources in Tarim Basin, Xinjiang Production and Construction Crops and Ministry of Science and Technology, Alar, China; ^4^ College of Life Science, Tarim University, Alar, China

**Keywords:** *Schizothorax biddulphi*, microsatellite, genetic diversity, genetic structure, genome survey

## Abstract

To protect the germplasm resources of *Schizothorax biddulphi*, we developed and used 20 pairs of polymorphic microsatellite primers to analyze the genetic diversity and structure of populations. A total of 126 samples were collected from the Qarqan River (CEC), Kizil River (KZL), and Aksu River (AKS) in Xinjiang, China. The results showed that 380 alleles were detected in 20 pairs of primers and the average number of alleles was 19.0. The effective allele numbers and Nei’s gene diversity ranged from 1.1499 to 1.1630 and 0.0962 to 0.1136, respectively. The Shannon index range suggested low levels of genetic diversity in all populations. The genetic distance between the CEC and AKS populations was the largest, and the genetic similarity was the smallest. There was a significant genetic differentiation between CEC and the other two populations. The UPGMA clustering tree was constructed based on population genetic distance, and the clustering tree constructed by individuals showed that the AKS population and KZL population were clustered together, and the CEC population was clustered separately. Also, the group structure analysis also got the same result. It can be seen that although the three populations of *S. biddulphi* do not have high genetic diversity, the differentiation between the populations was high and the gene flow was limited, especially the differentiation between the CEC population and the other two populations. This study not only provided genetic markers for the research of *S. biddulphi* but the results of this study also suggested the need for enhanced management of *S. biddulphi* populations.

## Introduction

*Schizothorax biddulphi*, in the order Cypriniformes and family Cyprinidae, is a symbolic species distributed only in the Tarim River system of Xinjiang Uygur Autonomous Region, China (Le and Chen, 1998; Luo et al., 2012). Due to its slow growth, late sexual maturity, low fecundity, and higher requirements for living environment, coupled with the destruction of the ecological environment and human interference in recent years, the habitat of *S. biddulphi* has been destroyed and continuously reduced, and the population has been drastically reduced (Nie et al., 2014). Therefore, *S. biddulphi* was listed in the China Red Data Book of Endangered Animals–Fish Volume in 1998 (Le and Chen, 1998) and in the Xinjiang Provincial Second-Class Protected Animals in 2004. In 2020, *S. biddulphi* upgraded to the national secondary protection fish of China.

Microsatellite DNA, also known as simple sequence repeats (SSRs) and short tandem repeats (STRs), is a nucleotide sequence consisting of 1–6 nucleotides in the repeat motif (Tautz and Renz, 1984). SSR has the advantages of large number, wide distribution, high polymorphism, high conservation, easy detection, high repeatability, and co-dominant characteristics, so it is widely distributed in eukaryotic genomes as a molecular marker (Li et al., 2008; Mohanty et al., 2016). SSR plays an important role in biological genetics research and has been increasingly used in conservation genetics research of endangered animals that lack genetic information, such as Rhesus macaque (*Macaca mulatta*), Asian elephant (*Elephas maximus*), Arabian oryx (*Oryx leucoryx*), and land snail (*Satsuma myomphala*), to guide the management and conservation of endangered animals (Li et al., 2012; Kang et al., 2017; Elmeer et al., 2019; Marasinghe et al., 2021; Qiu et al., 2021).

Most genetic diversity assessment methods are only applicable to diploid organisms, but studies showed that schizothoracine was polyploidy, mainly tetraploid, hexaploid, and octaploid (Wang et al., 2016; Guo et al., 2019). However, in most of the existing studies on *S. biddulphi*, the complexity of polyploidy was ignored, and the analyses followed the diploid approaches (Gong et al., 2012; Luo et al., 2012). This neglect might not explain population genetic diversity and genetic differentiation among populations more accurately. To overcome it, the most common solution was to convert microsatellite's polyploid genotypes to pseudodiploid genotypes, which allowed the use of many popular methods (Wang and Scribner, 2014; Liu et al., 2017). A few studies attempted to develop microsatellite primers for *S. biddulphi* to analyze genetic differences in populations based on polyploidy information, but the available markers were still very limited (Yang et al., 2014), and the polymorphism of these markers was low, which is difficult to revealing genetic differentiation among populations. Therefore, in this study, we wanted to develop more high polymorphic microsatellites for the research and population management of *S. biddulphi*. Also, three populations from the Tarim River Basin were investigated to provide support for the germplasm resource conservation of *S. biddulphi*.

## Materials and Methods

### Materials

**FIGURE 1:**
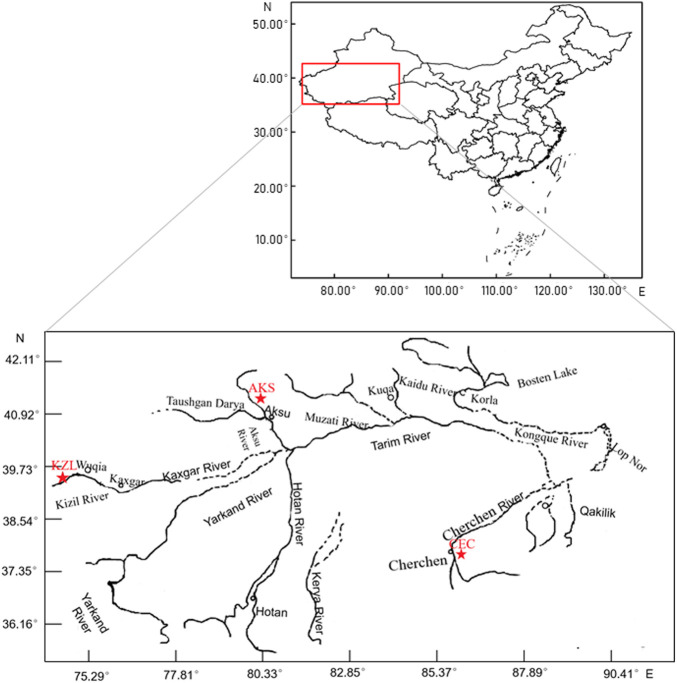
Population sampling sites of *Schizothorax biddulphi*. ★: each sampling point.

From July 2017 to July 2019, 126 tail fin samples were collected from 3 populations, including 44 from Cherchen River (CEC; 37°42′37″N, 85°41′45″E), 69 from Kizil River (KZL; 39°31′12″N, 75°14′20″E), and 13 from Akesu River (AKS; 40°14′4″N, 80°5′36″E), Xinjiang, China (Figure 1). All samples were stored in absolute alcohol and brought back to the laboratory and stored at −20°C. Total genomic DNA was extracted from the tail fin using the TIANamp Genomic DNA Kit (Tiangen, Beijing, China) following the manufacturer’s instructions. The concentration and quality of DNA were estimated using a DeNovix DS-11 spectrophotometer (DeNovix, Wilmington, United States).

### Genome Survey, SSR Locus Development, and Primer Design

The samples for the genome survey were randomly selected from 3 *S. biddulphi* from Cherchen River. Five DNA libraries of 270 bp(2), 350 bp(2), and 500 bp(1) insert size were constructed and sequenced by Illumina Hi-Seq 2000.

The Perl script MIcroSAtellite (MISA, http://pgrc.ipk-gatersleben.de/misa/misa.html) was used for genic-SSR markers with perfect repeat units of 2–6 nucleotides. The minimum SSR length criteria were defined as six reiterations for dinucleotide and five reiterations for other repeat units. About 288 microsatellite loci were selected, of which 288 with enough flanking sequence were used for primer design using Primer Premier 5.0 (Premier Biosoft International).

All SSR primer pairs were initially screened for a sample set comprising 42 randomly selected specimens, optimized for reproducible amplification using standard PCR conditions with annealing temperatures altered according to the primer sequences. By performing electrophoresis on an 8% non-denaturation polyacrylamide gel, 20 of 30 loci were successfully amplified and shown to be polymorphic, which were deposited in the GenBank (Accession Nos. MT211579–MT211598).

### SSR Genotyping

The forward primers for the 20 SSR loci were labeled with fluorescent dyes (6-FAM, HEX, TAMRA, and ROX). PCR amplification was carried out using genomic DNA extracted from 126 *S. biddulphi*. Each 50 μL PCR contained 2 μL genomic DNA, 25 µL RTaq DNA Premix, 1 µL forward and reverse primers, and 21 µL double-distilled water. The thermocycling conditions were as follows: initial denaturation for 5 min at 94°C, followed by 35 cycles of 30 s at 94°C, 30 s at the annealing temperature (Table 1), and 40 s at 72°C, and finally 10 min at 72°C. The PCR products were separated by capillary electrophoresis using an ABI3730xl DNA analyzer (Applied Biosystems) after confirmation of PCR amplification on a 1.5% agarose gel. The genotyping of polymorphism microsatellite locus analysis was performed using the software GeneMapper v4.0 (Applied Biosystems).

**TABLE 1:** 20 microsatellite locus characteristics of *Schizothorax biddulphi*.

Locus	Primer sequence (5′-3′)	Repeat motif	Tm (°C)	Product size (bp)	Accession
T43	F:TGTATGAAAGCACAATGGG	(TG)_5_(TA)_6_	48.0	164–191	MT211579
	R:GTGTAGTTTTTAGCGGCA				
T90	F:TTATGATTTTCTTTCGTC	(TA)_12_	49.0	339–349	MT211580
	R:TCAACATTCTCTCTTTTT				
T136	F:GCTCTTGCTTTCTCTGGG	(AC)_11_	50.8	316–358	MT211581
	R:GATTTCTCGTGCTGTCATT				
T144	F:TGAGGTTTGGTGTCTGTG	(ATGG)_7_	56.6	91–154	MT211582
	R:ATCCATCTGTCCGTCTGT				
T166	F:GGTGTCATCTGTTTTGTGG	(GT)_5_	50.8	217–262	MT211583
	R:CTGTATCCCTGGTTAGCG				
T175	F:GTATTCCTGTATCTCACCTG	(TG)_6_	48.0	239–322	MT211584
	R:TCTCATCCTCTTTCGTTC				
T218	F:TGCTGGCAGAGAATGAATGT	(ACT)_11_	53.5	341–405	MT211585
	R:TTGGTTGATGTCAGAGGTTG				
T227	F:CTTTTTGTTGCTGAGGTGTT	(CTA)_16_	49.0	272–372	MT211586
	R:TACTGTGAGGGATTTGTCGT				
T229	F:GTAAAACCAACAGCACAGCC	(CA)_22_	48.0	220–314	MT211587
	R:GGGACAAGGGGAAAAAACTA				
T230	F:TTTCATCCTTCTGCCACCAC	(AC)_16_	48.0	276–318	MT211588
	R:ATCCGCTAATCATAAACACC				
T231	F:TATTACAAAACGAATGGAGG	(AC)_25_	56.6	311–392	MT211589
	R:AAGAAACAAAGGTAGATCAA				
T239	F:AAAGAAGGAAGAAAAGCAGC	(TC)_20_	49.0	197–254	MT211590
	R:GGGGAAGGAAAAAAAGTGAC				
T246	F:GTATCTGGTCCATCTGCTCC	(GA)_15_	56.6	220–240	MT211591
	R:TCTGTCATGTTCTTTTGCTTT				
T255	F:CCTGAACAAATAAAACACCA	(TG)_20_	48.0	257–285	MT211592
	R:ACTCAAGGATAGACAAAACAT				
T259	F:TGGGTTTTAGTATTTTTGTC	(TA)_20_	48.0	351–372	MT211593
	R:ACTTTTTCTGTCTCGTTTCA				
T260	F:CTTTCAAACAGACAAGAGGA	(GA)_22_	48.0	218–258	MT211594
	R:AAACAGTTCAAGATGTAAATAAT				
T269	F:CAAACAAACCTCTACCCTCC	(CT)_19_	53.5	160–341	MT211595
	R:AAACAGTTCAAGATGTAAATAAT				
T272	F:TATGTTATTGATGTGTTGATT	(AATG)_14_	56.6	253–301	MT211596
	R:GTGCACCCTTTAGTTGTTAG				
T277	F:AATGCTTTCCTTTCTCAGCT	(ATCT)_15_	48.0	281–380	MT211597
	R:GTCCCTATTTTTTGTGGTTC				
T278	F:AGCATCTTCTCATACATTTT	(ATCT)_23_	48.0	188–285	MT211598
	R:GTGTCTTTATTTCTTGTCATT				

### Analysis of Population Genetic Diversity and Genetic Structure

This study referred to the method of Zhu et al. (2002) to analyze the electrophoretic patterns. The polymorphic bands amplified by each primer were counted, a band at the same migration position was marked as “1,” and no band was marked as “0.” According to the capillary electrophoresis pattern analyzed by Gene mapper 4.1, the standard for adopting the data is that the signal value is above 400, there is no interference of other spurious peaks, and the peak shapes from the same site are all similar. Finally, the original data matrix is established. Data editing and format conversion were performed in GenAlEx v 6.501 software.

Population genetic parameters such as the polymorphic percentage, allele number (*N*_*a*_), effective number of alleles (*N*_*e*_), Nei’s genetic distance (*H*), and Shannon’s Information index (*I*) were analyzed using GenePop v 4.14. Poptree V2 was used to establish an NJ clustering tree representing population genetic relationships based on allele frequency (Takezaki et al., 2010). Pcoa-genalex V6.5 analyzed genetic relationships among populations based on genetic distance (Smouse and Peakall, 2012). Structure 2.3.4 (http://pritch. bsd. uchicago. edu/) was used to analyze the genetic structure of the population, and the mixed model and frequency correlation model were selected. Online software Structure Harvest was used to estimate the theoretical population number according to the ΔK algorithm (Evanno et al., 2005).

## Results

### Genome Survey in *Schizothorax biddulphi*

The genomic DNA of *S. biddulphi* was used to construct two 270 bp libraries, two 350 bp libraries, and one 500 bp library, which were sequenced and filtered to obtain 165.34 Gb of high-quality data. The total sequencing depth was about 174 ×, the Q20 ratio of sequencing data was above 95.63%, and the Q30 ratio was above 90.48% (Supplementary Table SA1).

**FIGURE 2:**
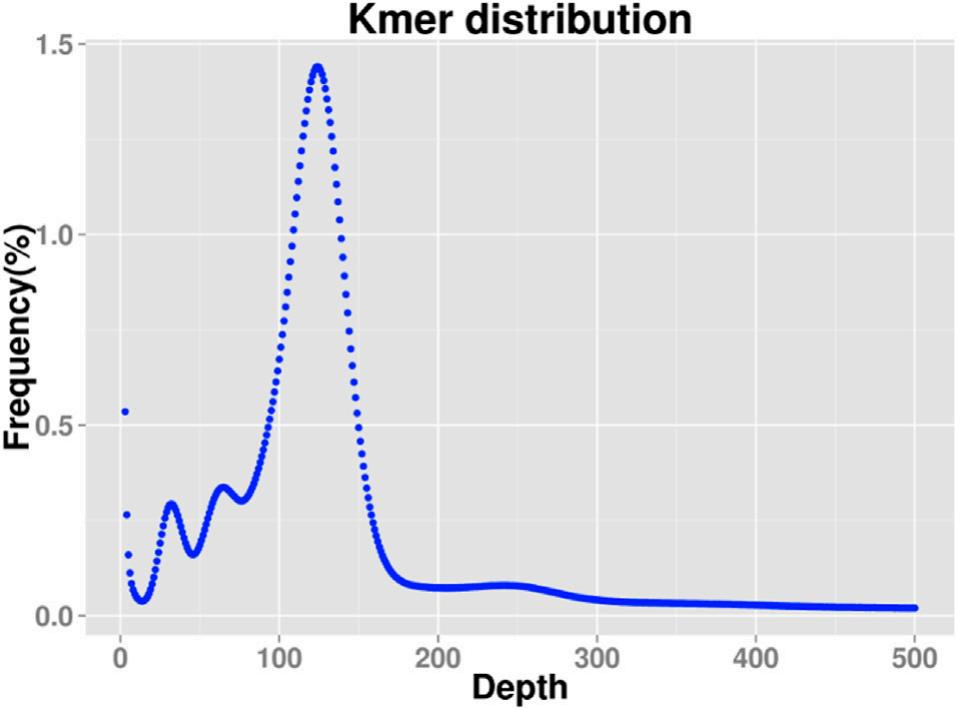
19 K-mer analysis for estimating the genome size of *Schizothorax biddulphi.*

The corresponding Kmer depth of the main peak is 120. There are three peaks in Kmer, the main peak, the 1/2 peak, and the 1/4 peak, suggesting that the species may be tetraploid (Figure 2). The total number of Kmer obtained from the sequencing data was 11,754,486,585. After removing the Kmer with abnormal depth, a total of 114,172,843,993 Kmer were used for genome length estimation, and the calculated genome length was about 947.30 Mbp. According to the Kmer distribution, the content of repeated sequences was estimated to be about 39.45%, and the heterozygosity was estimated to be about 0.81%. Therefore, the genome of this species belongs to a complex genome with high heterozygosity. Scaffold N50 was about 3.84 Kb and Contig N50 was about 869 bp (Supplementary Table SA2).

### Frequency Distribution of Different Types of SSR Markers

A total of 558,993 unigenes with a total nucleotide number of 1,125,446,683 were obtained from the genomic data of *S. biddulphi*. A total of 743,118 SSR sequences were detected, and 155,018 sequences contained more than 1 SSR (Supplementary Table SA3). The microsatellite-type richness of the detected *S. biddulphi* genome was high, and di-nucleotide repeat unit content was the most (42.74%), followed by mono-nucleotide repeat units (39.07%), and the content of tri-nucleotide (9.09%) and tetra-nucleotide (7.94%) repeat units was similar, the content of penta-nucleotide (0.92%) and hexa-nucleotide (0.24%) repeat units was less. The distribution density of the six nucleotide repeat types was positively correlated with the corresponding SSR content (Table 2).

**TABLE 2:** Distribution characteristics of SSR motifs in the genome of *Schizothorax biddulphi*.

Repeat type	SSR count	Percent	Base type	Main repeat motif	Density of SSR (per/Mb)
Mono-nucleotide	290,331	39.07	4	A/T,C/G	204.99
Di-nucleotide	317,627	42.74	12	AC/TG,CA/GT,TA/AT,TC/AG,GA/CT	133.01
Tri-nucleotide	67,536	9.09	59	AAT/TTA,ATT/TA, ATA/TAT,AAC/TTG	39.37
Tetra-nucleotide	59,001	7.94	7.94	TCTA/AGAT, GATA/CTAT, AGAC/TCTG	18.36
Penta-nucleotide	6,862	0.92	582	TATTA/ATAAT, AAAAT/TTTTA	3.64
Hexa-nucleotide	1,761	0.24	418	GTGTGA/CACACT, ATATAC/TATATG	0.51
Total	743,118	100	1290	—	399.88

*S. biddulphi* microsatellites include 4 different mono-nucleotide repeat microsatellite types, 12 different di-nucleotide repeat microsatellite types, 59 different tri-nucleotide repeat microsatellite types, 215 different tetra-nucleotide repeat microsatellite types, 582 different penta-nucleotide repeat microsatellite types, and 418 different hexa-nucleotide repeat microsatellite types. Among the mono-nucleotide repeats, A/T was the main base composition (93.23%). AC/TG (30.02%) was more in di-nucleotide repeat SSRs. AAT/TTA (29.44%) and ATT/TTA (23.57%) had high frequency of tri-nucleotide repeats. TCTA/AGAT and GATA/CTAT appeared more frequently in tetra-nucleotide repeats. TATTA/ATAAT and AAAAT/TTTTA appeared more frequently in penta-nucleotide repeats. GTGTGA/CACACT and ATATAC/TATATG appeared more frequently in hexa-nucleotide repeat microsatellite.

### Polymorphism of SSR Markers

Among the 288 pairs of primers, 30 pairs of primers with high polymorphism were screened by agarose gel electrophoresis and 8% non-denaturing polyacrylamide gel electrophoresis. According to the detection results of capillary electrophoresis, 20 pairs of SSR primers with high polymorphism were screened out from the above 30 pairs of primers (Table 1). The polymorphic parameters of the 20 primer pairs are shown in Supplementary Table SA4.

### Assessment of Genetic Diversity

A total of 380 alleles (*N*_*a*_) were detected, and the number of alleles per SSR locus ranged from 5 (T166) to 56 (T269), with an average of 19 alleles. The number of effective alleles (*N*_*e*_) ranged from 1.0595 (T269) to 1.5804 (T166), and the average number was 1.2517. Nei’s genetic distance (*H*) ranged from 0.0540 (T269) to 0.3449 (T166), with an average of 0.1635. Shannon’s Information index (*I*) for each SSR locus ranged from 0.1206 (T269) to 0.5188 (T166), with an average of 0.2773 (Table 3). In terms of population genetic diversity parameters, *H* and *I* were consistent, showing that the CEC was the highest and the AKS was the lowest (Table 4).

**TABLE 3:** 20 microsatellite locus genetic variation of *S. biddulphi*.

Locus	*N*_*a*_	*N*_*e*_	*H*	*I*
T43	7	1.5802	0.3425	0.5137
T90	7	1.3942	0.2281	0.3508
T136	11	1.2636	0.1788	0.2994
T144	17	1.1913	0.1324	0.2322
T166	5	1.5804	0.3449	0.5188
T175	8	1.5004	0.2901	0.4441
T218	11	1.1471	0.117	0.2181
T227	25	1.1217	0.0925	0.1754
T229	21	1.1549	0.1155	0.2111
T230	8	1.2647	0.1646	0.2685
T231	39	1.0946	0.0789	0.1593
T239	26	1.1727	0.1246	0.2243
T246	11	1.3715	0.2221	0.3488
T255	12	1.1983	0.1428	0.2522
T259	17	1.1857	0.1356	0.2437
T260	24	1.2362	0.1641	0.2801
T269	56	1.0595	0.054	0.1206
T272	17	1.2659	0.1784	0.2966
T277	34	1.1066	0.0919	0.1838
T278	24	1.1449	0.1081	0.2046

**TABLE 4:** Genetic diversity parameters of the six populations of *Schizothorax biddulphi*.

Population	Polymorphic	*N*_e_	*H*	*I*
	Percentage			
CEC	61.32	1.1615	0.1136	0.1936
KZL	66.84	1.1630	0.1084	0.1841
AKS	35.53	1.1499	0.0962	0.1529

The range of genetic differentiation index and gene flow was 0.107–0.224 and 0.866–2.086, respectively. The genetic differentiation index among CEC, AKS, and KZL was great genetic differentiation (0.15 < *F*_st_ < 0.25), and there was moderate genetic differentiation between AKS and KZL (0.05 < *F*_st_ < 0.15). The gene flow between AKS and KZL was the highest, while the gene flow between CEC and the other two populations was low (Table 5).

**TABLE 5:** Gene flow *N*_m_ (above diagonal) and *F*_st_ values for pairwise comparison (blow diagonal) among the three populations of *Schizothorax biddulphi*.

	CEC	KZL	AKS
CEC	—	0.897	0.866
KZL	0.218**	—	2.086
AKS	0.224**	0.107	—

** p< 0.001.

The range of Nei’s genetic distance and genetic identity between every two populations was 0.021–0.044 and 0.957–0.980, respectively. The genetic distance of Nei’s between CEC and AKS was the longest (0.044), and the genetic identity was the least. The Nei’s genetic distance between AKS and KZL was the closest (0.021), and the genetic identity was the largest (Table 6).

**TABLE 6:** Nei’s genetic distance D (above diagonal) and genetic identity (blow diagonal) among the three populations of *Schizothorax biddulphi*.

	CEC	KZL	AKS
CEC	—	0.038	0.044
KZL	0.962	—	0.021
AKS	0.957	0.980	—

### Genetic Structure and Genetic Relationship Among *Schizothorax biddulphi* Populations

**FIGURE 3:**
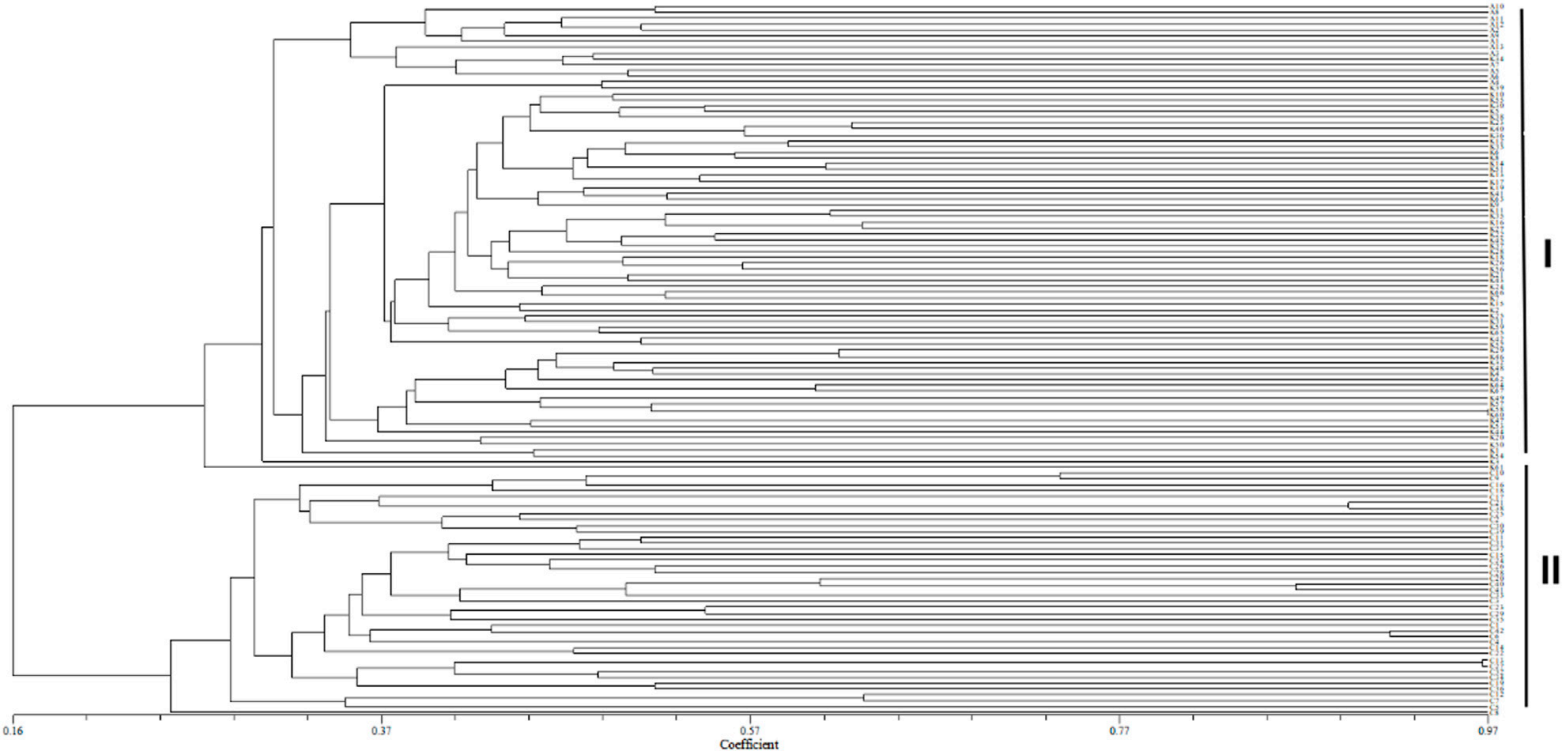
UPGMA clustering tree of 126 samples of *Schizothorax biddulphi*. I: all individuals of the Aksu and Kyzyl rivers; II: all individuals of the Qarqan River.

**FIGURE 4:**
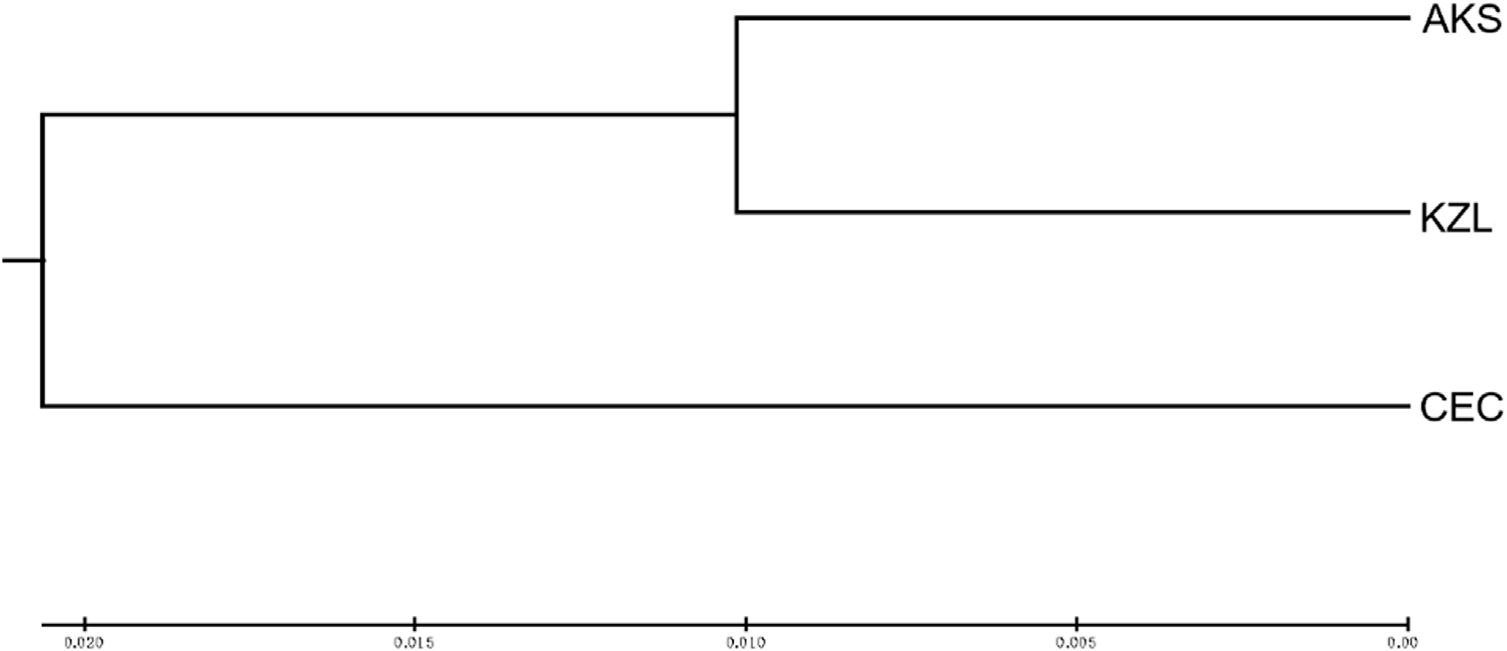
UPGMA clustering tree of 3 populations of *Schizothorax biddulphi*.

Cluster analysis was performed on an individual (Figure 3) and population (Figure 4) of *S. biddulphi*. The results showed that 126 samples of *S. biddulphi* were roughly divided into two clusters, with AKS and KZL individuals clustered into one cluster and CEC individuals clustered into another cluster. At the same time, the UPGMA tree constructed according to population genetic distance was consistent with the clustering tree constructed by individuals, the AKS and KZL clustered into a cluster, and the CEC clustered into a single cluster.

AMOVA results showed that 20% of the genetic variation of *S. biddulphi* came from inter-population and 80% of the genetic variation came from intra-population (Table 7). Therefore, the genetic variation within populations was larger than that between populations.

**TABLE 7:** Analysis of molecular variance (AMOVA) in three populations of *Schizothorax biddulphi*.

Source of variation	*d.f.*	Sum of squares	Variance components	Percentage of variation
Among populations	2	408.829	5.282	20
Within populations	123	2524.794	21.217	80
Total	125	2933.623	26.499	100

**FIGURE 5:**
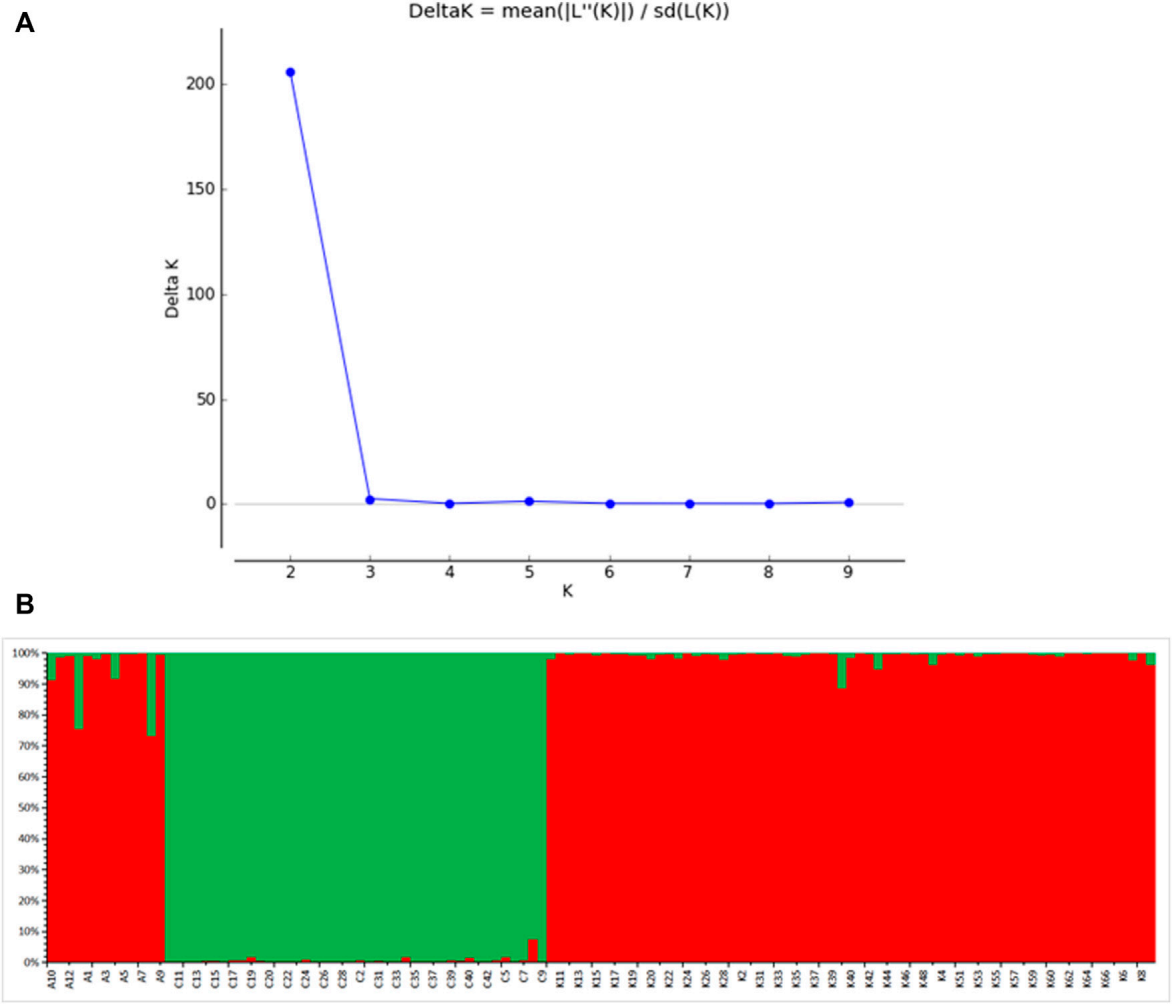
Model choice criterion lnP (D) of the structure analysis for each K value **(A)**; *K* = 2, structure analysis in all three *Schizothorax biddulphi* populations **(B)**, CEC (green), and other two populations (red).

Division of the dataset into two subclusters (*K* = 2) produced the best assignment of individuals to subclusters, and subsequent analysis of each subset determined the appropriate number of populations within each subset (Figure 5A). There are two gene banks in the three wild populations (Figure 5B). When *K* = 2, the gene composition of AKS and KZL samples mainly came from gene bank 1. The gene composition of CEC was mainly derived from gene bank 2 (Table 8).

**TABLE 8:** Proportion of ancestry of each population in three gene pools defined with the model-based clustering method.

*K* = 2	AKS	CEC	KZL
Gene bank 1	0.9415	0.0067	0.9909
Gene bank 2	0.0585	0.9933	0.0091

## Discussion

### SSR Locus Development of *Schizothorax biddulphi*

*S. biddulphi* is an economic fish only distributed in the Tarim River system of Xinjiang, China, and is endangered at present. However, there are few nucleotide sequences related to *S. biddulphi* microsatellites found in the GeneBank database, and the number of markers that can be used for population genetic diversity research is limited. Therefore, this study obtains microsatellite sequences through genome sequencing of *S. biddulphi*, and develops and screens out microsatellite markers with high polymorphism. SSRs in the genome of *S. biddulphi* are not only abundant but also more numerous, with a predominance of dinucleotide repeats. This is a common phenomenon in the most aquatic animal genome, such as *Takifugu rubripes* (Cui et al., 2006), *Scophthalmus maximus* (Ruan et al., 2011), *Ietalurus punetaus* (Serapion et al., 2004), *Fugu rubripes* (Edwards et al., 1998), and *Marsupenaeus japonicus* (Lu et al., 2017).

### Genetic Diversity of *Schizothorax biddulphi*

Genetic diversity parameters, such as the effective number of alleles, Nei’s genetic distance, and Shannon’s Information index, can be used to reflect the size of the genetic diversity; in a fixed parameter range, the greater the value of these parameters, the higher the gene richness of the population (Lu et al., 2017). In this study, the average number of alleles of 20 pairs of polymorphic primers in 3 populations of *S. biddulphi* was 19, which was all higher than 9.5, 4.9, and 3.5 of the studies of Gong et al. (2012), Yang et al. (2014), and Luo et al. (2012). However, there was a large difference between the number of alleles at the SSR loci and the number of effective alleles, which indicated that alleles were unevenly distributed in the populations (Kimura and Ohta, 1969). Compared with the polymorphic locus frequency of other related species such as *Schizopygopsis malacanthus* (68.22%), *Schizopygopsis malacanthus chengi* (66.36%), S*chizothorax wangchiachii* (61.21%), *Schizothorax griseus* (61.24%), *Schizothorax grahami* (60.63%), *Schizothorax dolichonema* (57.01%), and *Schizopygopsis stoliczkae* (57.48%), the genetic diversity of *S. biddulphi* is relatively lower (Meng and Zhang, 2007; He et al., 2012). Molecular variance analysis of genetic differences between populations showed that genetic variation was mainly from within populations, with small differences between populations, which was consistent with the result of the study by Yang et al. on Tashikuergan, Duolang headworks, Muzhati River, Yulongkashi River, and Kalakashi River populations (Yang et al., 2014). At the same time, the results of Nei’s genetic distance and Shannon’s Information index in this study were consistent, indicating that the genetic diversity of CEC was the highest among the three sampling sites, while that of AKS was the lowest. In a finite population, the number of generations required for accidental new selectively neutral mutations depends on the effective population size, and low-density populations favor the accumulation of selectively neutral mutations (Kimura, 1979; Barros et al., 2020). Therefore, the uneven distribution of alleles in the population of *S. biddulphi* may be caused by the decrease in population size. The dams built on the Tarim River in recent years have blocked the migration of fish, leading to a significant increase in the frequency of their inbreeding, which in turn causes low heterozygosity and genetic diversity of the offspring. If this situation is not effectively improved, the genetic diversity of the population will continue to decline (or the inbreeding coefficient will continue to increase), eventually resulting in an irreversible decline of the population and risk of extinction. The genetic variation obtained by microsatellite markers was similar among different populations of *S. biddulphi*, and the genetic variation mainly occurred within populations rather than between populations. CEC may retain the original population resources, or the result may be caused by the small number of AKS samples.

### Genetic Structure and Relationship of *Schizothorax biddulphi*

*F*_st_ and *N*_m_ are two important indicators to measure population genetic structure (Pierson et al., 2015). In this study, the genetic differentiation between AKS and KZL was moderate, while that between other populations in pairs was high. However, the size of gene flow between populations was the opposite; that is, there was more gene exchange between AKS and KZL than between other populations. This may be due to the Kizil River being an upstream tributary of the Kashgar River, which originates from the eastern parts of the Pamir Mountains (Hayward, 1869). The Kashgar River eventually flows eastward into the Tarim River, while the Aksu River is the main tributary of the Tarim River, so there is a wide range of gene exchange between the two populations (Wang et al., 2021). Although the Kashgar River has been separated from the mainstream of the Tarim River at present, the separation time is relatively short, and the resulting genetic differentiation is relatively small. The reason for the large genetic differentiation between CEC and the other two populations is as follows: As early as 220 BC, the Tarim River and Cherchen River converged in Lop Nur (Xinjiang, China), and the gene exchange between aquatic organisms in the two rivers was directly influenced by the evolution of Lop Nur (Haysa et al., 2016). Studies have shown that Lop Nur appeared in the Upper Miocene about 5 mega annum ago and dried up at the end of the Pliocene due to climatic reasons. Subsequently, the climate gradually improved and the dryness decreased during 2.7–1.5 mega annum ago, and the surface of Lop Nur recovered before 1.5 mega annum (Yuan and Yuan, 1998; Luo et al., 2006; Chang and Chang, 2013). The genetic differentiation between CEC and other populations may have been caused during the period of Lop Nur drying, which caused geographical isolation between CEC and other populations and the fragmentation of its habitat. Although Cherchen River and Tarim River intersect at Taitema Lake, the genetic differentiation between CEC and other populations still exists significantly. For aquatic organisms, geographical isolation barriers between different water areas usually lead to obvious population genetic differentiation, so the pattern of fish distribution often determines its genetic differentiation pattern (Bergek and Björklund, 2007; Riginos et al., 2011). For example, the Arctic grayling (*Thymallus arcticus*) in the Itkillik, Kuparuk, and Sagavanirktok basins in the foothills of the Brooks Mountains undergo altered dispersal and gene flow due to movement restrictions caused by river drying (Golden et al., 2021).

Based on Nei’s genetic distance, the UPMGA method was used for cluster analysis of three *S. biddulphi* populations and individuals. The results showed that AKS and KZL clustered into a cluster, and CEC clustered into a cluster. Structure cluster analysis also shows that the CEC population had great genetic differences from the other two populations, which further verifies the Cherchen River due to early break with the Tarim River, cutoff of the genes within or between is a kind of communication, increase the genetic distance, and then the genetic similarity between CEC and the other two populations was small. After 1950s, although the Kizil River also gradually lost its connection with the Tarim River, the time of geographical isolation between KZL and AKS was relatively short, so the genetic distance between the two populations was relatively close and the genetic similarity coefficient was high.

## Conclusion

In this study, 20 new SSR markers of *S. biddulphi* were developed by genome survey. These markers used were informative enough and could detect genetic diversity among *S. biddulphi*. The results of this study indicated that Cherchen River had preserved relatively primitive *S. biddulphi* population resources due to its early separation from the mainstream of Tarim River, and showed higher genetic diversity than the other two populations. However, the genetic diversity of *S. biddulphi* was still relatively low compared with other schizothoracin. If it is not its protection and intervention, *S. biddulphi* gene flow between populations appears greatly reduced or even cutoff and will lead to further genetic differentiation between populations, and make it into a vicious circle. Therefore, we must protect the genetic resources of *S. biddulphi* scientifically and rationally, strengthen the protection of its ecological water environment, and improve gene exchange between populations (Caballero and Toro, 2002, Jasim Aljumaili et al., 2018, Roques et al., 2016).

## Data Availability

The datasets presented in this study can be found in online repositories. The names of the repository/repositories and accession number(s) can be found below: https://www.ncbi.nlm.nih.gov/, PRJNA818042.
