# Supplementary material for: Analysis of Population Genetic Diversity and Genetic Structure of Schizothorax biddulphi Based on 20 Newly Developed SSR Markers
Source: Front Genet. 2022 Jun 13;13:908367. doi: 10.3389/fgene.2022.908367 (PMC9234283; doi:10.3389/fgene.2022.908367)
Supplement: Supplementary file 1 [file Table1.DOCX]

**Additional Tables**

**Table A1** Statistical table of sample sequencing results

| **Library** | **Data (Gb)** | **Depth (×)** | **Q20 (%)** | **Q30 (%)** |
| --- | --- | --- | --- | --- |
| 270 bp_1 | 34.42 | 36.33 | 95.97 | 90.91 |
| 270 bp_2 | 26.93 | 28.43 | 95.91 | 90.76 |
| 350 bp_1 | 37.88 | 39.98 | 95.63 | 90.48 |
| 350 bp_2 | 39.39 | 41.59 | 95.89 | 90.99 |
| 500 bp_1 | 26.72 | 28.20 | 96.91 | 93.10 |
| Total | 165.34 | 174 | -- | -- |

**Table A2** Statistical information on the Tarim schizothoracin genome

|  | **Scaffold number** | **Scaffold length (bp)** | **Scaffold**  **N50 (bp)** | **Scaffold**  **N90 (bp)** | **Scaffold**  **max (bp)** | **Gap total length (bp)** |
| --- | --- | --- | --- | --- | --- | --- |
| **Scaffold** | 558,993 | 1,125,446,683 | 3,841 | 730 | 88,414 | 59,614,722 |
| **Contig** | 2,198,989 | 1,065,831,961 | 869 | 221 | 44,792 | 36.70 |

**Table A3** SSR analysis results statistics

| **Searching item** | **Numbers** |
| --- | --- |
| Total number of sequences examined | 558,993 |
| Total size of examined sequences (bp) | 1,125,446,683 |
| Total number of identified SSRs | 743,118 |
| Number of SSR containing sequences | 285,598 |
| Number of sequences containing more than 1 SSR | 155,018 |
| Number of SSRs present in compound formation | 193,269 |

**Table A4** Genetic diversity analysis of locus

| Loci | *N*_a_ | *N*_e_ | *H*_e_ | *I* |
| --- | --- | --- | --- | --- |
| T43 | 3 | 1.266 | 0.209 | 0.363 |
| T90 | 4 | 1.553 | 0.302 | 0.450 |
| T136 | 5 | 1.205 | 0.153 | 0.272 |
| T144 | 9 | 1.188 | 0.135 | 0.240 |
| T166 | 4 | 1.606 | 0.348 | 0.517 |
| T175 | 6 | 1.267 | 0.185 | 0.313 |
| T218 | 6 | 1.172 | 0.145 | 0.272 |
| T227 | 20 | 1.213 | 0.149 | 0.263 |
| T229 | 16 | 1.175 | 0.139 | 0.257 |
| T230 | 6 | 1.196 | 0.151 | 0.272 |
| T231 | 20 | 1.203 | 0.150 | 0.265 |
| T239 | 22 | 1.220 | 0.167 | 0.294 |
| T246 | 6 | 1.504 | 0.318 | 0.492 |
| T255 | 7 | 1.263 | 0.159 | 0.336 |
| T259 | 13 | 1.317 | 0.207 | 0.338 |
| T260 | 18 | 1.349 | 0.234 | 0.382 |
| T269 | 13 | 1.267 | 0.194 | 0.332 |
| T272 | 16 | 1.397 | 0.250 | 0.397 |
| T277 | 17 | 1.198 | 0.159 | 0.291 |
| T278 | 22 | 1.227 | 0.172 | 0.302 |
